# Dynamic Models Identification for Kinematics and Energy Consumption of Rotary-Wing UAVs during Different Flight States

**DOI:** 10.3390/s23239378

**Published:** 2023-11-24

**Authors:** Krzysztof Falkowski, Michał Duda

**Affiliations:** 1 Avionics Department, Institute of Aviation Technology, Faculty of Mechatronics, Armament and Aerospace, Military University of Technology, 00-908 Warsaw, Poland; 2 Doctoral School, Military University of Technology, 00-908 Warsaw, Poland; michal.duda@wat.edu.pl

**Keywords:** dynamic models, identification, UAV, simulation

## Abstract

This article presents the method of identifying dynamic models for different flight states of a rotary-wing UAV for simulations. Experimental flights with real-life UAVs were conducted to obtain data necessary for identification. Dynamic models were identified with time series methods performed using Matlab R2022b software. Such models can later be implemented in simulations to represent the behavior of real-life objects. Simulation is the first stage of developing a real-life UAV system, where prototyping with physical models is problematic. Therefore, obtaining accurate models is crucial for the simulation process to be reliable. Presented methods do not require knowledge of UAV construction, and complex mathematical equations do not need to be derived. Also, verification of obtained models was performed to make sure that they were identified correctly. In particular, the presented method was proven effective and successfully used in some applications.

## 1. Introduction

Interest in applications of quadrotor drones is rising rapidly due to the development of robotic technologies. Scientists worldwide propose and research new control algorithms that can be implemented to execute specific tasks or improve existing solutions. Many of them are theoretical dissertations not transferred to practical applications. The research provided in this paper focuses on an attempt to simulate a real-life UAV with all its physical parameters.

Deployment of a real-life UAV system requires many robots to be built, programmed, and launched. The time and cost of those processes can grow significantly, especially for testing and prototyping [1]. This makes simulations the first complex system preparation stage [2]. Implementing a virtual system allows for conducting a series of tests with different conditions using only a PC. Such tests are not dependent on the weather, do not require robot maintenance, and can be terminated if any malfunction appears [3]. Prototyping an algorithm for maneuvering real-life UAVs can be dangerous for the surroundings and the robot [4].

Most of the research focuses on control algorithms, a set of rules that defines how robotic agents propagate throughout the area. Tests using virtual simulations provide the mathematical output of the robot’s behavior, and often, their physical parameters are either not addressed or simplified. The more precise the data implemented in the simulation, the more realistic its output is. In the case of simulating a UAV’s behavior, not only the commanding algorithm matters but also the dynamics of its movement. Simulation of a UAV requires knowledge of its physical parameters and dynamics. Every neglection or simplification at such an early research stage can lead to incorrect conclusions, affecting further real-life applications [5,6].

In the case of practical applications, energy consumption is a crucial factor in the usage of autonomous robots, especially UAVs [7]. The energy used to perform the task should be addressed in the research, as UAVs depend highly on their battery life. A power source defines their performance, such as velocities, altitude, capacity, etc. [8,9].

A specific field that is significantly based on simulations and modeling is formation flights and swarms of UAVs. Due to the numerous agents required to perform, it is much easier to model and simulate them virtually rather than prepare the same number of physical objects. The literature provides a significant amount of research regarding multi-agent systems, where such systems are modeled. As shown by Huang, a second-order consensus algorithm can resolve collision avoidance in formations [10]. UAV formation obstacle avoidance can be performed using an artificial potential field and a consensus algorithm [11]. The trajectory of flight for every agent in the formation can be planned before starting the mission [12]. Even a mission of tracking a moving target with a formation of UAVs can be modeled and simulated, as proven in [13]. Although such research proved correct in all these cases, it is based on mathematical equations and analytical contemplation. Such an approach is time-consuming. Also, it requires precise knowledge of UAV internal parameters, or simplifications are assumed that ignore the physical aspect of the agents. The next step of every theoretical research should be implementation in real-life systems, which is often not addressed. Our study presents a method for obtaining models for UAV flights based on experimental data that can later be implemented into simulations of single-object or multi-agent systems. Such an approach ensures the model’s compatibility with a physical object and is more applicable in practical solutions. The conflict in Ukraine showed that UAV systems can be prototyped from available UAVs that are not necessarily made for military purposes. An ability to rapidly identify models of such objects might come in handy in many military applications, especially when time is essential.

Similar studies regarding preparing dynamic models for UAVs have already been performed and described in the literature. As shown in [14], dynamic models of UAVs can be derived mathematically, including specific factors such as weather conditions, flying speed, and payload. This approach requires complex mathematical methods and knowledge of the modeled object [15]. The other approach assumes that it is possible to collect experimental data using real-life objects and subsequently identify models with it. As presented in [16], creating energy consumption models for UAVs for their different flight states is possible. Such research can also be extended to find and impact factors such as climb rate or desired altitude [17]. All such methods prove their efficiency. Our study used similar identification methods, extending them with different flight stages, particularly models for horizontal acceleration and deceleration, which could be used in modeling collision avoidance—a critical feature of every aerial vehicle system.

This paper presents a practical approach to identifying dynamic models for rotary-wing UAVs. Experimental flights were performed, which allowed the collection of flight data for the UAVs and thus helped to improve the precision of data used in simulations. Such an approach permitted performing simulations close to the actual behavior of UAVs without solving complex mathematical formulas regarding quadrotors. This was especially useful when the consumption of energy was calculated. Also, the method proved to be quicker and relatively simple and focused on physical systems over theoretical contemplations that often cannot be transferred straight into real-life applications.

## 2. Materials and Methods

The main goal of the presented research was to build a control system for a UAV. The study was divided into four stages as follows:

1. Prepare a simulation program executing a desired algorithm.
2. Conduct experimental flights with real-life UAVs to collect flight data for the identification of dynamic models.
3. Identify dynamic models of UAVs in examined flight stages.
4. Implement obtained dynamic models into the simulation. Conduct tests and gather and analyze results.

All study stages were described in more detail in the other parts of this paper.

### 2.1. Physical Parameters in Simulations of UAVs

Before launching a real-life system, the first step was to test it in virtual simulations. This stage required models of the objects to be implemented into the simulation as a representation of real-life objects. The more accurate those models were, the more realistic the simulation output was.

**Figure 1:**
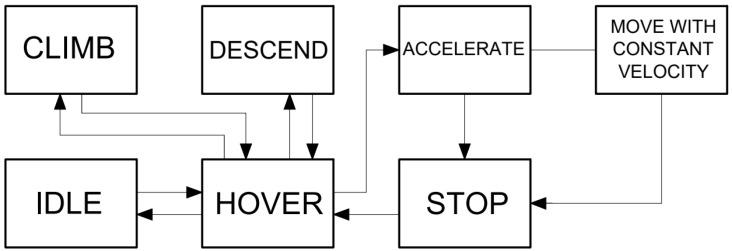
Diagram of flight states.

Knowing the drones’ dynamic models was vital in conducting the simulations for two reasons. Firstly, it allowed us to correctly measure the time and energy consumed by the UAVs while performing the mission. Those two factors were significant for the optimization of the performance. Drones operated in different flight stages during the simulation, as presented in Figure 1. During all of them, they consumed energy at different rates, and knowing those rates enabled the calculation of total energy consumption. UAV systems are known for their relatively low battery capacities, and optimizing the performance of drone systems according to energy consumption is one of the main factors to include in research. Secondly, the time required to complete transition states (accelerating, climbing, etc.) and maximal velocities enabled us to derive the total time needed to finish the task.

The second reason for identifying dynamic models of the drones was the safety and fluency of their movement. Due to the implementation of the collision avoidance feature, knowing the distance required to accelerate or decelerate was necessary because it was vital for the path planning of the drones. The distance covered during stopping was crucial as the algorithm needed to compute when the drones had to start decelerating to avoid a collision if such a situation appeared.

### 2.2. Experimental Flights and Data Collection

The mathematical models can be estimated by the identification method. This method uses time series models such as ARX, AR, and ARMAX. The transfer function can be estimated from the ARX model:

(1) $$Y\left(z\right)=G\left(z\right)U\left(z\right)+H\left(z\right)E\left(z\right),$$

where $Y\left(z\right)$—the Z transform of an output signal, $U\left(z\right)$—the Z transform of an input signal, $E\left(z\right)$—the Z transform of the white noise, $G\left(z\right)=\frac{B\left(z\right)}{A\left(z\right)}$—a transfer function in terms of *z* operator, and $H\left(z\right)=\frac{1}{A\left(z\right)}$—a color filter in terms of *z* operator. $A\left(z\right),\;B\left(z\right)$—polynomials in terms of *z* operator.

The transfer function of an identified model can be converted from a digital model to an analog model. The continuous transfer function is equal:

(2) $$G\left(s\right)≅{\left.G\left(z\right)\right|}_{{forT}_{s}},$$

where $T_{s}$—the sampling period.

**Figure 2:**
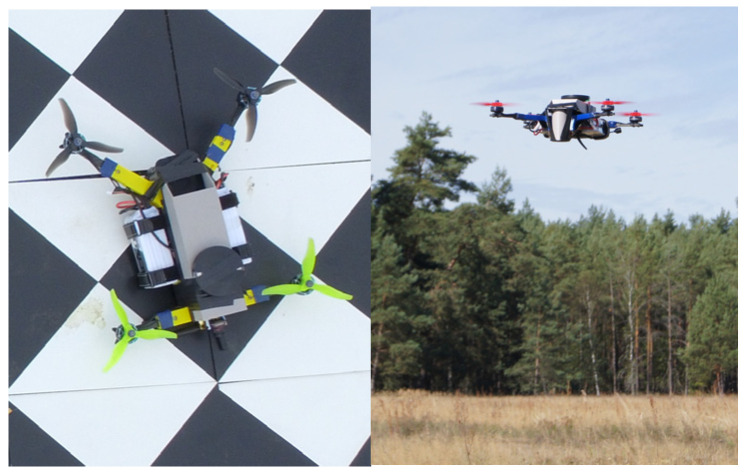
The UAV used for identification tests.

The authors of the stochastic recovery path simulation need a model of take-off, landing and cruise flight, acceleration, and deacceleration of the UAV. We assume that the UAVs will not change altitude at the operational zone. The UAV quadrotor was used to conduct the identification experiment. The quadrotor is presented in Figure 2.

**Figure 3:**
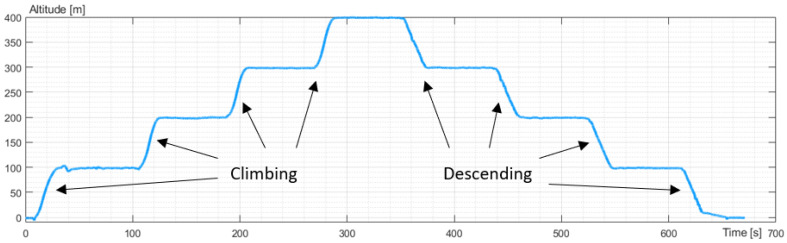
The profile of altitude recorded during the experiment.

The experiments were divided into the vertical and horizontal movement of the UAV. The vertical move study included take-off to an altitude of 100 m. Next, the altitude increased to 200 m, 300 m, and 400 m and decreased to 300 m, 200 m, and 100 m. The last point of the study was landing. The UAV hovered at each point of the study. It took the same position (latitude and longitude), and the flight controller stabilized and corrected the angular orientation of the UAV. The altitude flight is presented in Figure 3.

**Figure 4:**
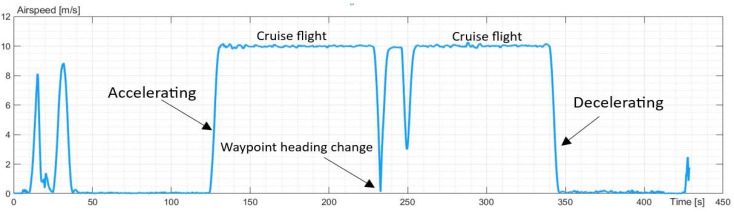
The profile of airspeed recorded during the experiment.

The next experiment applied to horizontal movement. The UAV took off to an altitude of 200 m and moved to the waypoint. There, the UAV changed the heading and returned to the point of take-off, and the UAV landed. The UAV had a defined latitude, longitude, and altitude of the take-off and waypoint. The maximum airspeed was 10 m/s, as defined in the flight plan. The airspeed profile is presented in Figure 4.

The mechanical and electrical parameters were recorded during identification experiments. The voltage, current, airspeed, vertical speed, altitude, latitude, longitude, and reference airspeed and altitude were recorded. The experiment enabled us to obtain the transfer function between the reference value and energy. The transfer functions were estimated for the take-off, climbing, descending, cruise, and landing of the UAV.

## 3. Results

### 3.1. Identifying Dynamic Models

When experimental data were collected, it was possible to identify dynamic models for every flight state. The models were identified according to a section of gathered data corresponding to the same state in actual flight. Then, to verify it, dynamic models were compared to a suitable part of data from other flights. Finally, the entire flight was simulated using identified models mimicking the real-life test, and it allowed us to compare the total energy consumption and scale of errors.

Identification was performed in Matlab R2022b. Input data were control signals from the flight controller, and output data were registered flight parameters. Identified parameters were a change of power over time, vertical displacements, and horizontal velocities over time during every considered flight state. The dynamic models were presented as a continuous time transfer function.

The only difference was hover, which was considered a static state; hence, dynamic model identification could not be performed for it. Knowing that it depended on height [9], the power was calculated as the average power required to hover at a given altitude. Then, knowing the result for a few different altitudes, the dependence of power on time was approximated with function. According to Rotratu [18], the power required to hover is given by:

(3) $$P=\frac{T^{\frac{2}{3}}}{\sqrt{2\rho A}}$$

where T—total thrust force, A—total area swept by rotors, and ρ—air density.

Assuming that during hover, T and A are constant, power depends on air density, which in International Standard Atmosphere [19] can be approximated as a reversed exponential proportional to flight altitude H. In this case, the power required to hover can be written as:

(4) $$P=K\;\cdot H^{z}$$

where $K=\frac{T^{\frac{2}{3}}}{\sqrt{2A}}=const,\;$ z—proportion exponent, and H—flight altitude.

Using collected data, the equation describing power required to hover on flight altitude H was approximated with:

(5) $$P\left(H\right)={119.49H}^{0.02}\;[W]$$

#### 3.1.1. Vertical Movement Models

**Figure 5:**
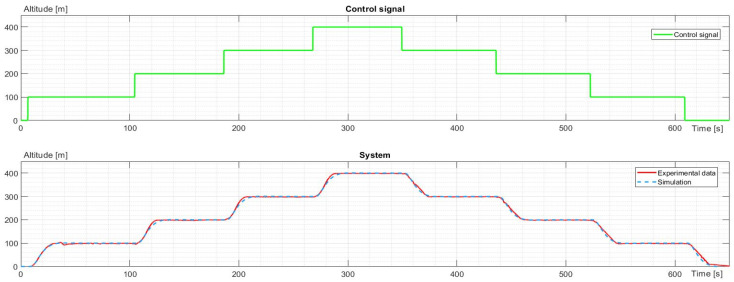
The control signal and characteristic comparison of experimental data and simulation for altitude.

The first dynamic models to be identified were vertical movements: ascending and descending. The data for the process were four subsequent climbs by 100 m from 0 to 400 m for ascending. Analogically, there were four subsequent decreases by 100 m from 400 m to 0 for the descent. Models for the altitude changes were identified together for both states, and models of power were created separately. A comparison of experimental data and identified models for altitude change is shown in Figure 5.

As seen in Figure 5, the simulated characteristic matches the experimental data; hence, the simulation can be assumed to mimic the vertical movement of the drone with sufficient precision. The following transfer function describes the identified model:

(6) $$G_{1}\left(s\right)=e^{-2.9s}\frac{0.03071}{s^{2}+0.2923s+0.03071}$$

**Figure 6:**
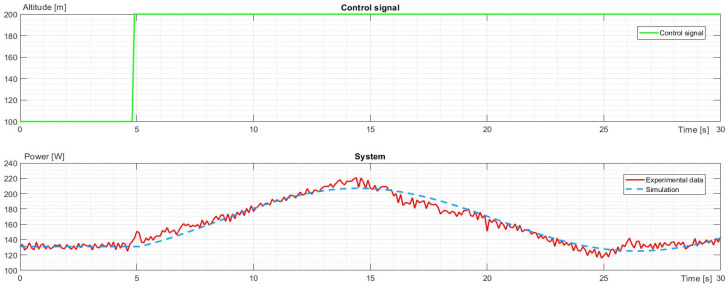
The control signal and characteristic comparison of experimental data and simulation for power change during climbing.

**Figure 7:**
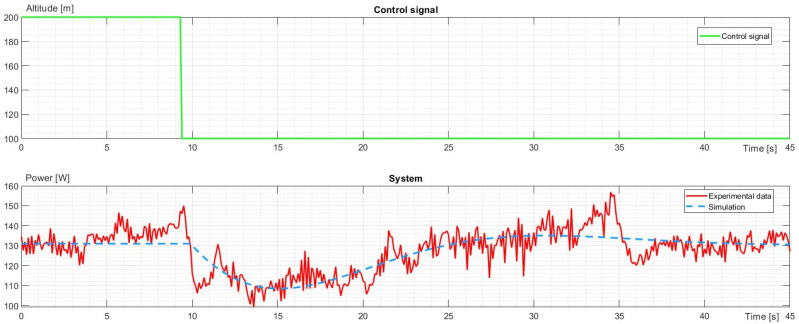
The control signal and characteristic comparison of experimental data and simulation for power change during descending.

The second part of the identification was made for the power required to climb and descend to a 100 m difference in altitude. As the system was assumed to be linear, offsets were brought to 0, so as not to break homogeneity and superposition principles. Then, it was added to the stable state power required for hovering. The power characteristics for climb and descent are shown in Figure 6 and Figure 7.

As can be seen, at the beginning of the climb, the power rapidly increased as the drone had to accelerate upwards. At some point, the UAV reached sufficient speed to reach the desired altitude, requiring less and less thrust. Hence, the power started to decrease. At the climb’s final stage, the drone had to lose gathered speed using the force of gravity. At that point, the power dropped below a stable state shortly after stabilizing to the value required to hover. The identified model for power during the climb is presented below:

(7) $$G_{2}\left(s\right)=e^{-0.2s}\frac{0.07023s+0.02383}{s^{2}+0.006887s+0.06957}$$

When the drone needed to descend, power first dropped, and the reduced thrust became lower than the force of gravity, making the drone accelerate downwards. At some point, the power rose to slow the rate of descent. Subsequently, the thrust had to grow over the force of gravity for some time to lose downward velocity and stabilize the flight to hover at the desired altitude. The transfer function for power during descent is presented below:

(8) $$G_{3}\left(s\right)=e^{-0.5s}\frac{0.09489s-0.00001339}{s^{2}+0.228s+0.05344}$$

In both cases, the experimental data was distorted by noise that could not be seen in the simulated response. Optical evaluation allowed us to conclude that the signal trends were similar in both cases, yet further validation of the models had to be conducted.

#### 3.1.2. Horizontal Movement Identification

**Figure 8:**
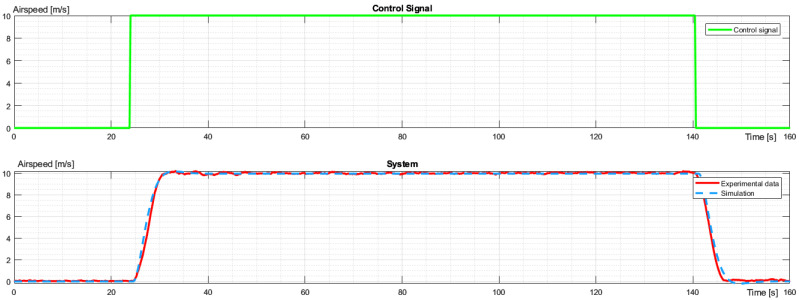
The control signal and characteristic comparison of experimental data and simulation for airspeed during horizontal flight.

Analogical identification was performed for horizontal movement. The flight states were accelerating, moving with constant velocity, and decelerating (stopping) as presented in Figure 8. The energy consumption had linear dependence on acceleration and flying with constant velocity. While accelerating, the temporal power grew proportionally to velocity, and during the constant speed movement, the energy consumption settled around a constant value. This value was calculated as an average taken from the segment where the drone flew with constant velocity. The identified model for horizontal flight is presented below:

(9) $$G_{4}\left(s\right)=e^{-0.6s}\frac{0.03569}{s^{2}+0.935s+0.3588}$$

**Figure 9:**
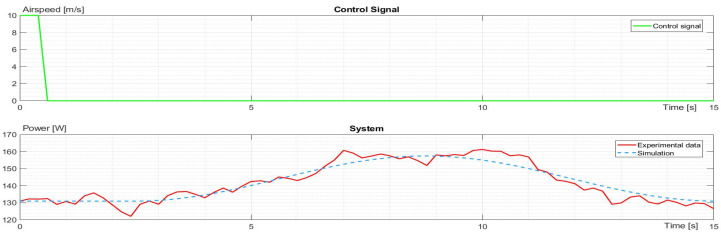
The control signal and characteristic comparison of experimental data and simulation for power during deceleration.

When the drone was commanded to stop, the desired velocity was instantly changed to 0, so the UAV not only aborted the flying forward but also tried to stop in the shortest time. Hence, it rotated backward and created counter thrust to its movement direction, creating backward force. The power change for deceleration is shown in Figure 9.

As can be seen, after being commanded to stop, the power consumption rapidly increased as the drone started the backward rotation and increased the thrust. Then, it rotated forward, decreasing the power to settle in a hover. Below, the identified transfer function for power change during deacceleration is presented:

(10) $$G_{5}\left(s\right)=e^{-0.6s}\frac{0.02583s-0.3294}{s^{2}+1.905\cdot 10^{-10}\;s+0.2492}$$

Knowledge of dynamic changes in airspeed (9) provided the information required to simulate the collision avoidance feature in the systems. For example, the distance needed to stop the UAV can be derived from the model. Also, the movement dynamics allowed us to forecast further positions of the drones and predict conflicting configurations. Also, whenever a UAV is obligated to stop, it consumes additional energy that can be calculated using the identified model (10).

### 3.2. Model Verification

After identifying the dynamic models, the next step is their verification. This is required to prove that identified models represent different cases. The other experimental flight was considered, and its flight plan was recreated in simulation. Next, the experimental and simulated data were compared.

**Figure 10:**
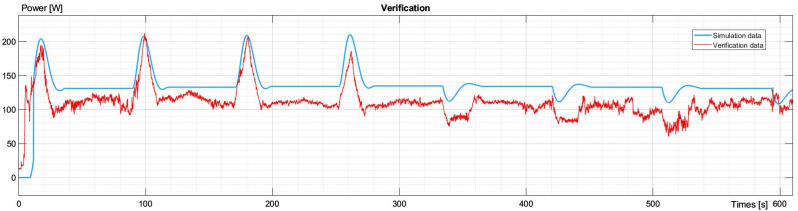
Characteristic comparison of experimental data and simulation for power change during the entire flight for verification.

As seen in Figure 10, the nature of both characteristics was the same. Flat segments and peaks were synchronized and had a similar magnitude. Yet, the charts could have been more perfectly aligned as their height was mainly defined by the constant power required to remain at altitude. This value depends on many factors such as atmospheric conditions, winds, etc., so if they were different in compared attempts, the average power required to hover also differed.

Nevertheless, such comparisons proved that most of the identification was performed correctly. It also showed how vital the power required to hover is for UAVs and how volatile that variable is.

## 4. Discussion

This article describes a method for identifying dynamic models of UAV flight. As shown in this paper, obtaining dynamic models of certain maneuvers of quadrotor robots is possible without deriving a complete mathematical model, which is complex for such objects. Properly conducted experimental flights with data recording are required to perform identification.

This method is useful, especially regarding energy consumption and temporal power during flight stages. Battery capacity is one of the most significant challenges of present UAVs, and energy characteristics are integral to mission planning. The presented method allows us to identify the power characteristics of flying UAVs quickly.

In the case of rotary-wing UAVs, the most critical flight state is remaining at a constant altitude. During a hover or while performing maneuvers, a significant part of the generated thrust has to be directed down to counteract the force of gravity. The presented results show that the gross amount of temporal energy had to be consumed so the robot would not fall. Therefore, correctly identifying this power is crucial in preparing a UAV flight model.

Identified models for certain flight states can be implemented in virtual simulations of the UAV. This way, simulations become more realistic while remaining in the virtual process. Kinematic models allowed us to mimic the movement of the UAVs, which helped us reproduce the acceleration and stopping distances, which are crucial in path planning and collision avoidance. Power parameter models allow us to derive energy consumption for the entire flight.

Simulating and deriving the power characteristics throughout the entire flight of the UAV allows for calculating the total energy that must be consumed to fulfill the planned mission. Such a study improves the path-planning process with another factor, which is especially significant for rotary-wing UAVs. Thanks to that, it is possible to optimize the total energy consumption of the system.

## 5. Conclusions

The identification method can be useful for studies on UAVs when researchers want to avoid deeply investigating UAV construction and the relations between power consumption and component structure. This method allows us to bypass complex mathematical methods, but it requires a real object to be available for experimental data collection.

Power consumption is a crucial parameter for every system based on UAVs, especially rotary-wing robots. Methods that allow us to estimate energy consumed in a mission beforehand help to better prepare for it. It is especially visible in swarm systems, where estimated performance and errors are multiplied by the number of actors in the swarm.

Presented validation data showed a misalignment of experimental and simulated data, which was a difference of a constant value over the entire chart. The validation data could have been gathered in different atmospheric conditions, or other unidentified factors influenced the results. This shows how rotary-wing UAVs are susceptible to various factors and noise. On the one hand, it shows how important it is to collect experimental data in ideal conditions; on the other hand, in real-life applications, conditions will probably not be perfect, and this should also be included in simulations.

In further studies, models will be implemented in simulations of swarms performing the Stochastic Coverage Task. Using acquired models should allow us to estimate the total energy and time required to complete the mission, and information on acceleration and deceleration (braking) will be useful for motion planning and collision avoidance.

## Data Availability

Data are contained within the article.
